# The More We Search, the More We Find: Discovery of a New Lineage and a New Species Complex in the Genus *Asparagopsis*

**DOI:** 10.1371/journal.pone.0103826

**Published:** 2014-07-30

**Authors:** Laury Dijoux, Frédérique Viard, Claude Payri

**Affiliations:** 1 Institut de Recherche pour le Développement (IRD), UR227 CoRéUs-LabEx-CORAIL, Noumea, New Caledonia; 2 Sorbonne Universités, Université Pierre et Marie Curie (UPMC) Univ Paris 06, UMR 7144, Station Biologique de Roscoff, Roscoff, France; 3 Centre National de la Recherche Scientifique (CNRS), UMR 7144, Divco team, Station Biologique de Roscoff, Roscoff, France; Griffith University, Australia

## Abstract

In the past few decades, in the marine realm in particular, the use of molecular tools has led to the discovery of hidden taxonomic diversity, revealing complexes of sister species. A good example is the red algal genus *Asparagopsis*. The two species (*A. armata* and *A. taxiformis*) recognized in this genus have been introduced in many places around the world. Within the nominal species *A. taxiformis*, previous molecular analyses have uncovered several lineages, suggesting the existence of sister species or subspecies. Although the genus has been well studied in some regions (e.g., the Mediterranean Sea and Hawaii), it remains poorly investigated in others (e.g., South Pacific). Our study mainly focused on these latter areas to clarify lineages and better determine lineage status (i.e., native vs. introduced). A total of 188 specimens were collected from 61 sites, 58 of which had never been sampled before. We sequenced the DNA from samples for three markers and obtained 112 sequences for the chloroplastic RuBisCo spacer, 118 sequences for the nuclear LSU rRNA gene, and 174 for the mitochondrial spacer cox2-3. Phylogenetic analyses using all three markers suggested the existence of two cryptic sister species with the discovery of a new clade within *A. armata*. This clade was found only in Western Australia, Tasmania and New Zealand, and is thus restricted to a subregional biogeographic unit. We also discovered a new, fifth lineage for *A. taxiformis* restricted to the South Pacific and Western Australia. Except for this newly described lineage, all other lineages showed a global distribution influenced by introduction events. These results illustrate the difficulty in accurately defining cosmopolitan species. Our findings also highlight the need for targeted (i.e., in poorly studied areas) and geographically extensive sampling efforts when studying taxa that have been introduced globally and that are likely to hide species complexes.

## Introduction

Cosmopolitan species — defined as species with a global distribution or spanning several biogeographic provinces — are common in the marine realm [1]–[3]. However, based on molecular data, seemingly cosmopolitan species often prove to be a complex of cryptic species (i.e., species morphologically indistinguishable despite being biological species with a divergent evolutionary history [4]). Transport by humans (i.e., biological invasions) may also artificially widen a species range and thus generate a cosmopolitan distribution [5], [6]: today, many species ranges go beyond natural barriers because human activities promote their transport and successful settlement far from their native range. For example, aquaculture activities represent a major vector for introducing marine species outside their common range at the global scale (e.g., the Japanese kelp *Undaria pinnatifida* [7] or the tunicate *Molgula manhattensis* [8]). Distinguishing between the relative importance of cryptic diversity and introduction to explain the large distribution of some taxa is not always straightforward, particularly for cryptogenic species (*sensu* Carlton [9], i.e., a species for which it is uncertain whether they are native or introduced because of their long-term association with human activities).

The genus *Asparagopsis* (Rhodophyta) is a good candidate for exploring these processes, namely cryptic diversity and introduction to explain a cosmopolitan distribution. The distribution of *A. armata* and *A. taxiformis* is broad and has been shown to be partly due to several introduction events [10], [11]. Within the genus *Asparagopsis*, eight nominal species have been reported, of which only two are currently retained excluding synonyms: namely *A. armata* Harvey and *A. taxiformis* (Delile) Trevisan [12]–[14]. *A. armata* was first described in Western Australia [15] and is also naturally present in New Zealand [16]. It is known as having been introduced to the northeastern Atlantic and Mediterranean Sea around the 1920s [17], [18], presumably from Southern Australia; the vector is unknown. *Asparagopsis taxiformis* was described by Delile in 1813 (as *Fucus taxiformis*; [19]) from a floating specimen collected near the lighthouse in Alexandria (Egypt, Mediterranean Sea), excluding the hypothesis of a Lessepsian migration (i.e., introduction from the Red Sea through the Suez Canal) at this date. However, its widespread presence today in the Mediterranean may well be explained by introductions [10] from other areas [20]–[23]. Molecular studies of *A. taxiformis* show that there are several cryptic lineages in this species, increasing uncertainty on its taxonomical status and biogeography. Andreakis et al. [3] described four evolutionary different lineages that may be indicative of at least two cryptic species: Lineage 1 (L1) is found in the Pacific, L4 in the Indo-Pacific; L2 is found in the Indo-Pacific, the Mediterranean Sea and North Atlantic; and L3 is found in the western Atlantic, the Canary Islands and eastern Mediterranean. While the taxonomic status of *A. taxiformis* is debated, studies on *A. armata* thus far have not shown any hidden molecular diversity revealing cryptic lineages [3], [10], [11], [24].

In the Mediterranean Sea, the genus is considered as being one of the “100 worst invasives” and described as “monospecific coverages, dominating many algal assemblages” [23], [25]–[27]. In Hawaii [11], *A. taxiformis* is also known to have been introduced, but formal studies on its environmental impact have not been conducted. In Europe, *A. armata* is more commonly studied as an ‘invasive species’ than *A. taxiformis*; this is likely due to the earlier introduction of the former compared to the latter (1923 and 1993, respectively [18], [28]). Altogether, there are no published data clearly linking either the ‘native’ vs. ‘non-native’ status or the ‘proliferative’ vs. ‘non proliferative’ status of *Asparagopsis* spp. to any particular habitat or species assemblage. Both species are found in the intertidal and shallow subtidal zones on hard substrates [21] on sheltered to exposed coasts (e.g. for *A. armata*: in its Australian native range [29] and in its Spanish introduced range [30], [31]). The main difference between the two taxa is climatic preference: temperate seas for *A. armata* and warmer seas for *A. taxiformis* (warm temperate to tropical regions).

Natural and human-mediated dispersal can play a role in the spread of the two taxa. Both have a haplo-diplontic, heteromorphic life cycle with alternating haploid gametophytic and diploid sporophytic stages. In addition to clonal gametophytic propagation in the two species, the gametophytes of *A. armata* can attach to various surfaces by their hooked branches [32], possibly favoring its spread in its introduced range [18]. These natural dispersal vectors are however unlikely to explain the cosmopolitan distribution of the two taxa. Several vectors of human-mediated transport have been suggested such as oyster farming [33] or maritime traffic (but see Flagella et al. 2007 [34] and Mineur et al. 2006 [35]: no *Asparagopsis* spp. have been recorded in surveys of ballast waters or ship hulls). In addition, they are directly exploited by humans: *A. taxiformis* has cultural value and has long been used for food in Hawaii [36] and *A. armata* is farmed in its introduced range (Northern Europe) to extract bioactive molecules [37].

**Figure 1:**
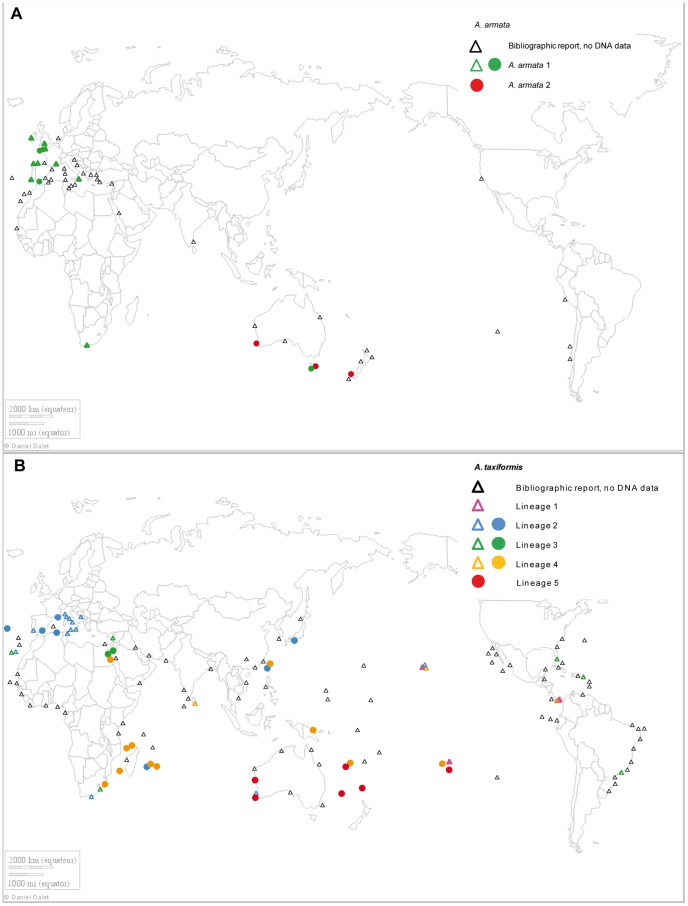
Present-day reported distribution of the currently recognized species (A) *A. armata* (A) and (B) *A. taxiformis*. Black symbols stand for morphological identification alone. Colored symbols are used to indicate different mitochondrial molecular lineages when known. Blank triangles report bibliographic data and filled circles indicate data obtained from the present study [3], [13].

Figures 1a and 1b depict present-day reports of the taxa described as *A. taxiformis* and *A. armata*. Some of these reports are not associated with taxonomic studies and misidentification between the two taxa may have occurred. Despite existing inventories and previous detailed studies (Figure 1, see Table S1 for details and references), only a limited amount of molecular data is available. Data tend to be restricted to some regions with very little information on the Indo-Pacific region, even though this region has been recognized as a likely diversification center [38]–[40]. The maps in Figure 1 show that some areas were overlooked. In particular, in New Caledonia only one individual has been sampled in the Southwest lagoon (2002, Passe Mato). Also, in a number of cases, reports from the Indo-Pacific are not associated with molecular identification, so that doubts persist as to the taxa and lineages currently present in the area. For example, Catala reported in 1950 the presence of *A. armata* in New Caledonia based on Valerie May's identification [41], but in the absence of herbarium vouchers, this identification cannot be confirmed. Likewise, in the southwestern Indian Ocean where *A. taxiformis* has been reported [22], morphological or molecular data are lacking.

Increasing sampling intensity and geographical coverage may reveal new lineages or indicate human-mediated spread of *Asparagopsis* taxa. The objectives of this study were three fold. First, to run genetic analyses on samples obtained from areas poorly represented in existing distribution maps, with a special focus on New Caledonia. Previous studies have examined one specimen only shown to belong to the L2 lineage [3]. Secondly, to assess the consistency of the observed lineages over time by comparing samples collected from 2001 to the present. Third, to determine which lineage is responsible for episodic *Asparagopsis* bloom events, recorded in New Caledonia from time to time since 1981 (department of scientific diving operations SEOH IRD), especially considering that L2 is known to be invasive in other regions (e.g., in the Mediterranean Sea). This study was based on the joint analysis of three markers chosen in the nuclear (LSU rRNA gene), mitochondrial (cox2-3 spacer) and chloroplastic (RuBisCo spacer, rbcL) compartment as each of them has different properties in terms of inheritance and mutation rates.

## Materials and Methods

### Study specimens

We obtained and analyzed a total of 188 specimens (17 identified as *A. armata* and 171 as *A. taxiformis*). Samples were collected from 61 sites within 20 biogeographic provinces [40] (Table 1 and Table S1). In New Caledonia, sampling permits were issued by local authorities in Province Nord, Province Sud and Province des Loyautés; in Papua New Guinea, the Department of Environment and Conservation of Papua New Guinea issued the permit; in Tasmania, permits were issued by the Department of Primary Industries, Parks Water Environment Wild Fisheries Branch; in Western Australia and Lord Howe Island, the Department of Environment and Conservation (WA) and the Department of Environment, Climate Change and Water (NSW) respectively issued the permits. For the other locations, no specific permit was required (studies did not involve endangered or protected species). The majority of samples were obtained from the tropical Indo-Pacific region, for which less molecular data is available in the literature and databases. Following collection, the specimens were dried on a paper towel, wrapped in filter paper and stored in silica gel. Samples from Taiwan were preserved in ethanol.

**Table 1:** Specimens sampled for this study across marine biogeographic provinces (name and definition provided in Spalding et al. [40]).

	Province	number of samples
*A. armata*	Mediterranean Sea	5
	Northern European seas	5
	Southwest Australian shelf	2
	Southeast Australian shelf	4
	Southern New Zealand	1
*A. taxiformis*	Agulhas	6
	Lord Howe and Norfolk Islands	5
	Lusitanian	10
	Mediterranean Sea	16
	Northern New Zealand	4
	Red Sea	6
	South China sea	4
	Southeast Polynesia	15
	South Kuroshio	4
	Southwest Australian shelf	1
	Tropical Southwestern Pacific	61
	Warm temperate Northwest Pacific	2
	West central Australian shelf	3
	Western Coral Triangle	5
	Western Indian Ocean	29

Details on sampling and additional data used for the analyses (i.e., sequences obtained from GenBank database) are provided in Table S1.

To check for temporal changes in the presence vs. absence of lineages in New Caledonia, 17 herbarium vouchers kept in the IRD-NOU phycological herbarium at IRD (*Institut de Recherche pour le Développement*) in Noumea were also added to the study. These samples were collected at the South lagoon of Grande Terre (Passe Mato 2002; Kué 2004), Koumac (2004), Lifou (2004), Touho (2004), Isle of Pines (BIODIP, 2005), Kié (2005), Bourail (2007), East lagoon of Grande Terre (CORALCAL1, 2007) and Chesterfield (CORALCAL2, 2008).

We retrieved and analyzed all molecular data available in GenBank for the two target species. We selected a total of 103 sequences to complement the geographic coverage of our study: for *A. armata*, 13, 1 and 9 sequences for the cox2-3 spacer, the rbcL spacer and LSU, respectively, and for *A. taxiformis*, 63, 2 and 15 sequences, respectively. Details on the origin of the specimens in each province and ecoregion and GenBank accession numbers are given in Table S1.

### DNA extraction and molecular analyses of collected specimens

For all newly collected material (i.e., except herbarium specimens), extractions were done with the DNeasy Plant minikit (Qiagen) or with the Nucleospin 96 Plant kit (Macherey Nagel) using 5 to 10 mg of dried material. To avoid extracting PCR-inhibiting polysaccharides, we did not incubate the specimens at 65°C prior to extraction.

For the herbarium specimens, DNA extraction was carried out using a CTAB protocol. This involved crushing 5 to 10 mg of dried material and adding 1 mL of CTAB buffer (2% CTAB, 1.4 M NaCl, 0.2% β-mercaptoethanol, 20 mM EDTA pH 8, 100 mM Tris-HCl pH 8, 0.1 mg/mL proteinase K) with 1 µL of proteinase K (0.1 mg/mL) to the mixture. Samples were then incubated in an agitated water bath at 60°C for 3 h and centrifuged for 10 min at 13 200 rpm at 4°C. The supernatant was collected and an equal volume of chloroform: isoamyl alcohol 24∶1 was added. The mixture was then shaken and centrifuged at 13 200 rpm for 10 min at 4°C. The supernatant was pipetted into fresh tubes and DNA was precipitated by adding isopropanol (2/3 of supernatant volume) and transferred to −20°C for 45 min followed by a centrifugation step at 13 200 rpm for 10 min. The pellet was washed with 500 µL 75% ethanol and centrifuged 10 min at 13 200 rpm. The supernatant was discarded and the pellet dried and dissolved in 50 µL of water.

The chloroplastic rbcL spacer [42], nuclear marker LSU [43] and mitochondrial marker cox2-3 spacer [44], were amplified following the protocol described in Andreakis et al. [10] with three modifications. First, LSU primers were redesigned for the study based on preliminary sequencing of a few specimens: LSU-At_F 5′-CGGGAAGAGCCCAACATG-3′ (Forward) and LSU-At_R 5′ CGGGTACCAGCACAASTGC-3′ (Reverse). Reactions were performed in a total volume of 25 µL containing 2 µL of extracted DNA diluted to 1∶50, 0.4 µM of forward and reverse primers, 1.25 U Taq polymerase (Jump Start Red Taq, Sigma) and no BSA. Second, 2 mM of MgCl_2_ and 0.25% of DMSO were added for all analyses involving LSU. Third, PCR cycles conditions were slightly modified: for the rbcL spacer and cox2-3 spacer markers, 5 touch-down cycles were added with annealing temperatures from 53°C to 48°C and 50°C to 45°C respectively; LSU annealing temperature was modified to 55°C for 1 min; and all extensions were performed at 72°C for 90 sec.

The quality of the PCR products was checked on a 1% agarose gel. PCR products were then sequenced by Macrogen (Macrogen Inc., Seoul, Korea) using the BigDye TM terminator method.

### Sequence analyses

Sequences obtained were aligned using BioEdit [45] and CodonCode (CodonCode Corp., Dedham, MA, USA) against sequences retrieved from the GenBank database.

Phylogenetic trees were inferred in MEGA5 (NJ, MP, UPGMA; [46]) or Seaview v4.4 (ML; [47]) using neighbor joining (NJ), maximum parsimony (MP), unweighted pair group method with arithmetic mean (UPGMA) and maximum likelihood (ML). For ML analyses, the model of nucleotide substitution was estimated using Findmodel (available at http://www.hiv.lanl.gov/content/sequence/findmodel/findmodel.html). Support for nodes was assessed by the bootstrap method [48] using 1000 replicates for NJ and MP analyses and 100 replicates for ML analyses. *Asparagopsis armata* was used as an outgroup. Other members of the Bonnemaisoniaceae family were also tested as outgroups (e.g., *Delisea pulchra*), but no difference in topology was observed.

For the cox2-3 spacer, several lineages were observed and distances between them (expressed as a number of base pair differences over the length of the sequence) were computed using DNAsp version 5 [49]. To check for consistency in molecular divergence across markers, distances were computed for the two other markers by grouping specimens according to their mitochondrial lineage.

Bayesian analyses were also performed using Beast with a GTR+G prior (10 million generations) and a strict clock model to calibrate the phylogeny. We used the divergence rate of cox2-3 spacer (5.2–6.1.10^−3^ substitutions.site^−1^.million years ago (Ma)^−1^) as a calibration to date the separation between each clade [3], [50].

Considering that the study specimens are members of a species complex or subspecies complex, haplotype networks were also computed for the cox2-3 spacer, the most polymorphic marker and the one showing the largest number of lineages. Haplotype networks are useful for revealing reticulations within a subsample of taxa that are closely related [51]. A non-hierarchical graphic representation can be plotted from these reticulations, and this type of network is more appropriate when reproduction events among closely related taxa are still occurring or when the time elapsed since divergence is short. Also, in the case of a complex history of multiple introductions, as in the *Asparagopsis* genus, networks can highlight dispersion and migration events [52]. Haplotype networks were computed using Network software applying the median joining algorithm (fluxus-engineering.com, [53]).

## Results

### Overall molecular diversity

A total of 112 sequences of 264 base pairs (bp) were obtained for rbcL spacer, 118 sequences of 606 bp for LSU and 174 sequences of 316 bp for the cox2-3 spacer (see Table S1 for details).

Over the whole dataset (and the two study taxa), the cox2-3 spacer displayed high molecular diversity with 59 haplotypes and 31.47% of polymorphic sites over a total of 278 specimens (from this study and GenBank data). This contrasts with the results obtained using the other two markers: the rbcL spacer was the least genetically diverse with only 7 haplotypes and 12.3% of polymorphic sites for 115 specimens; and the LSU marker showed 17 haplotypes with 15.95% of polymorphic sites for a total of 142 specimens.

### Phylogenetic analyses and time divergence

**Figure 2:**
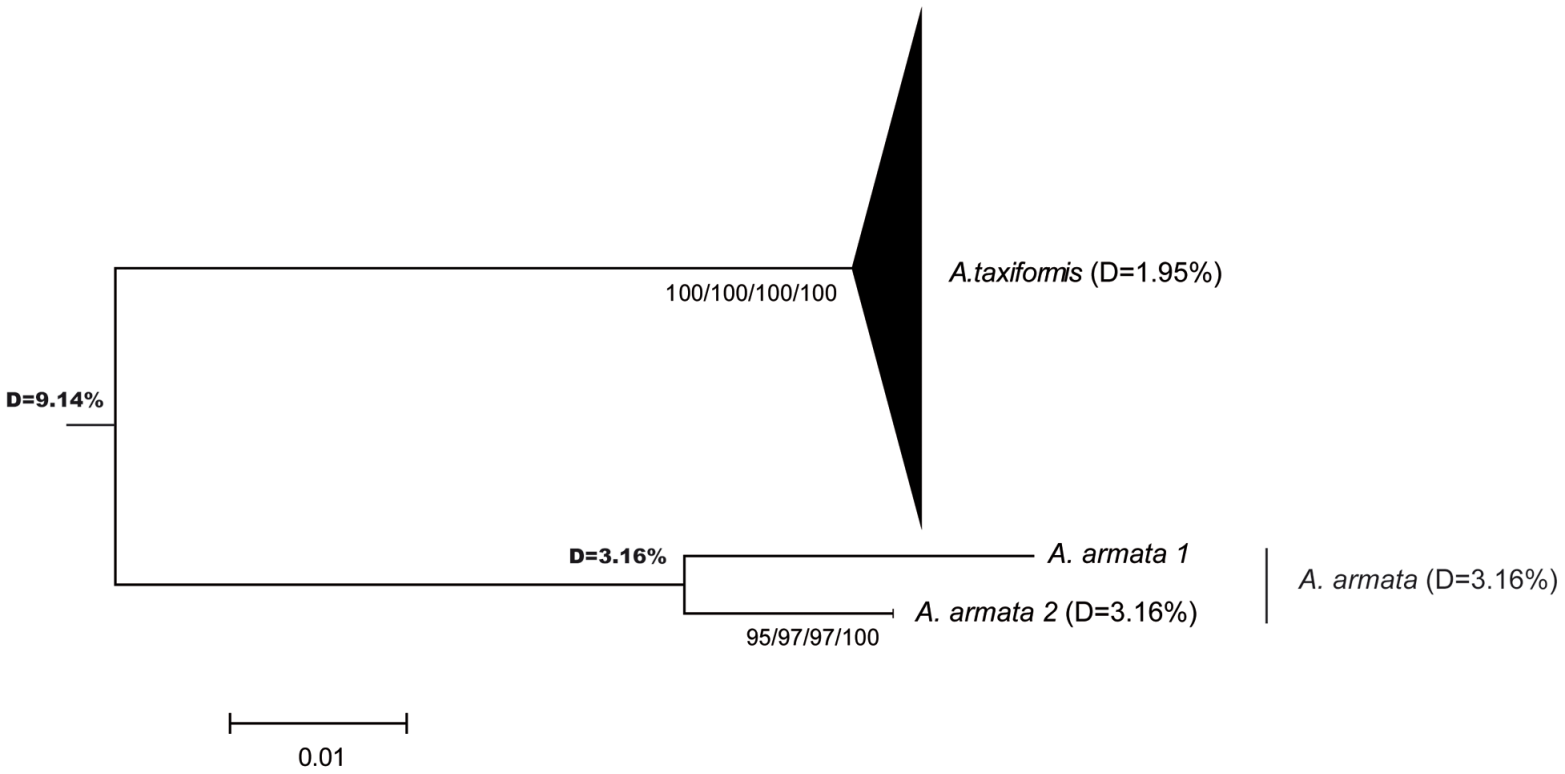
Neighbor-joining (NJ) tree for the rbcL spacer. Bootstrap values for NJ, MP, ML and UPGMA analyses, respectively, are given.

**Figure 3:**
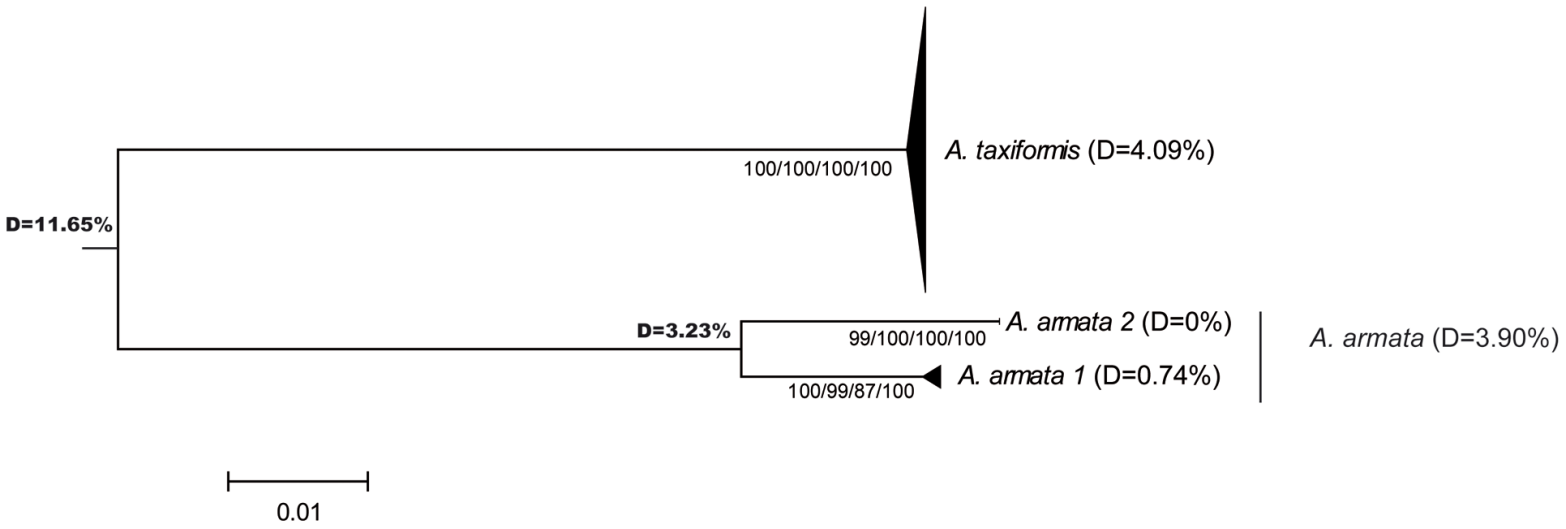
Neighbor-joining (NJ) tree for LSU. Bootstrap values for NJ, MP, ML and UPGMA analyses, respectively, are given.

**Figure 4:**
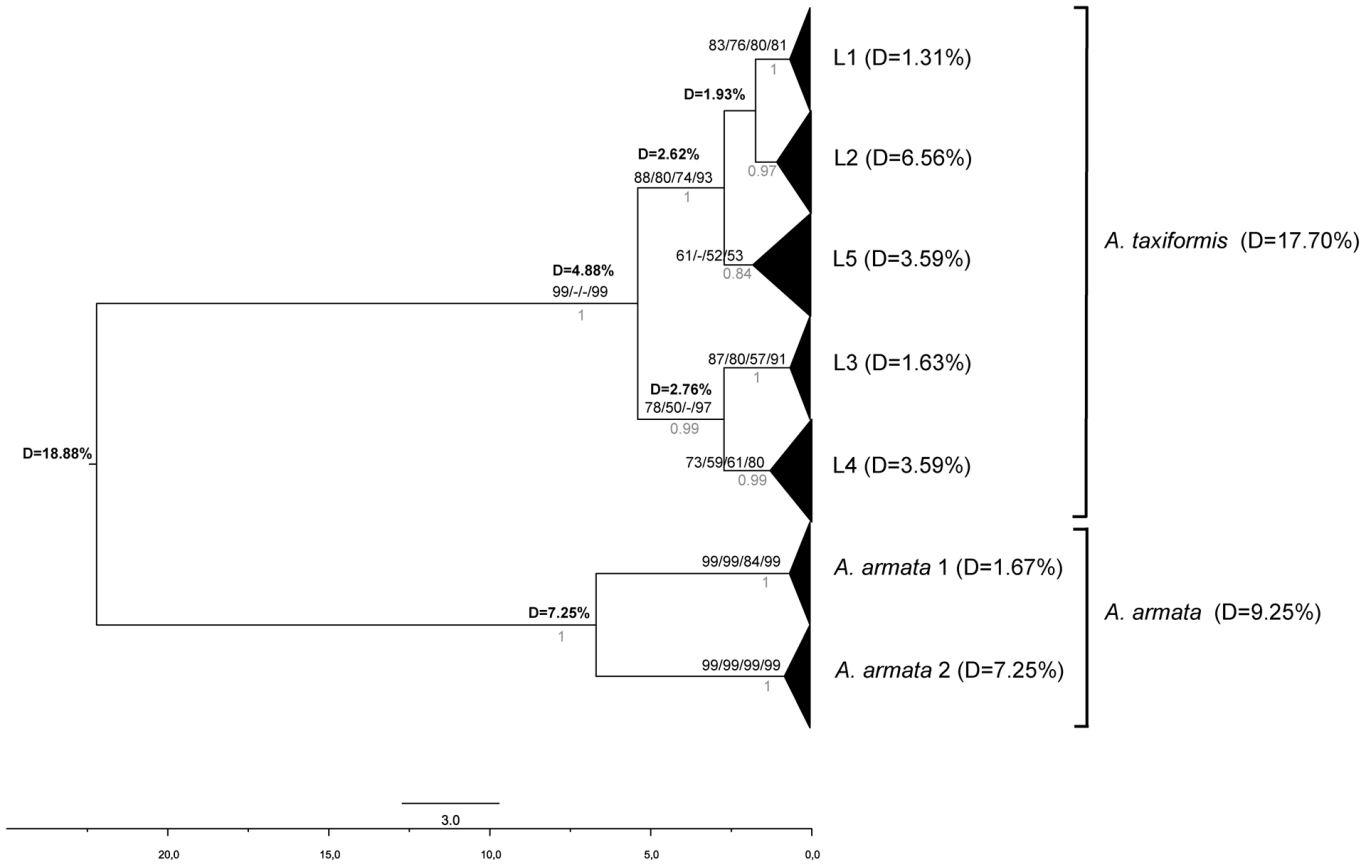
Neighbor-joining tree and Bayesian analysis for the cox2-3 spacer. Bootstrap values for NJ, MP, ML and UPGMA analyses, respectively, are given as well as posterior value from Bayesian analysis (values shown in light gray).

All phylogenetic reconstruction methods (NJ, MP, ML, UPGMA and Bayesian) produced similar tree topologies. Figures 2 to 4 show phylogenetic trees obtained by the NJ method for the three markers, with bootstrap values for NJ, MP, ML and UPGMA analyses for the rbcL spacer and the LSU marker plus posterior values obtained with a Bayesian analysis for the cox2-3 spacer. For the sake of clarity, the positions of the individual sequences in a given lineage are not presented (for the detailed trees, see Figures S1, S2, S3 for cox2-3, rbcL and LSU, respectively). As expected, we observed high molecular divergence between *A. taxiformis* and *A. armata* for each marker: the divergence ranged from 9.14% for rbcL spacer to 18.88% for the cox2-3 spacer (Figures 2 to 4).

In *A. armata*, two unexpected, well-supported, highly divergent and mutually monophyletic lineages were observed, not only with the cox2-3 spacer, but also with the two other markers (Figures 2, 3 and 4). They will be referred to as ‘clades’ to differentiate them from ‘lineages’. The latter are defined as a group of haplotypes clearly clustered in phylogenetic trees, but with lower level of divergence and usually not supported by all three markers. *A. armata* clade 1 grouped sequences from GenBank obtained from specimens collected in South Africa, France, the United Kingdom, Portugal and Spain together with sequences obtained in this study from individuals collected in Tasmania. *A. armata* clade 2 is composed only of sequences newly obtained from specimens collected in Western Australia, Tasmania and New Zealand for this study.

Within *A. taxiformis*, divergences were 1.95% for the rbcL spacer, 4.09% for LSU, but reached 17.70% for the cox2-3 spacer (see Figures 2 to 4). In *A. taxiformis*, five lineages were obtained for the cox2-3 spacer thus adding a new lineage to the four previously described lineages (L1 to L4; [3], [11], [54]). Divergences between these lineages are given in Table 2. Two groups were clearly distinguished based on the phylogenetic tree (Figure 4), one of which clusters lineages L1, L2 and L5 and one that clusters with lineages L3 and L4. These two clusters were not clearly observed with the rbcL spacer and LSU (Figures S1 to S3), which can be partly explained by their lower polymorphism compared to the cox2-3 spacer. This inconsistency can be highlighted by computing divergences for LSU and the rbcL spacer among specimens grouped according to their mitochondrial lineage (Table 2): divergences among the groups were sometimes larger than divergences within these groups. However, some phylogenetic relationships were consistent across markers, for instance L2 and L5, closely related according to the cox2-3 spacer, also show very low divergence with LSU and the rbcL spacer (Table 2).

**Table 2:** DNA divergence (number of base pair differences over the length of the sequence) within (on the diagonal) and between (below the diagonal) lineages (L1 to L5) computed for each marker.

cox2-3 spacer					
	L1	L2	L3	L4	L5
L1	1.31				
L2	1.93	6.56			
L3	5.37	4.95	1.63		
L4	4.99	4.66	2.76	3.59	
L5	3.33	2.37	4.7	5.05	3.59

rbcL spacer					
	L1	L2	L3	L4	L5
L1	-				
L2	-	0			
L3	-	1	1.56		
L4	-	0.55	0.93	1.17	
L5	-	0.01	1	0.55	0.78

LSU					
	L1	L2	L3	L4	L5
L1	-				
L2	-	3.23			
L3	-	0.43	0		
L4	-	0.28	0.18	0.51	
L5	-	0.16	0.38	0.22	0.68

Lineages were defined according to the mitochondrial marker cox2-3 spacer (for comparison with results by Andreakis et al. [3]; see Figure 3 for lineage definitions). ‘-’ stands for no data.

Assuming a divergence rate for the cox2-3 spacer of 5.2–6.1 10^−3^ substitutions.site^−1^.Ma^−1^(according to Zuccarello and West [50]), the time of divergence between L1 and L2 was found to be approximately 1.75 Ma. L5 was separated from L1 and L2 by about 2.72 Ma. L3 and L4 diverged from each other around 2.73 Ma and the two groups L1-L2-L5 versus L3-L4 diverged around 5.4 Ma (Figure 4).

### Network analysis and geographical distribution of the mitochondrial lineages in *A. taxiformis*

**Figure 5:**
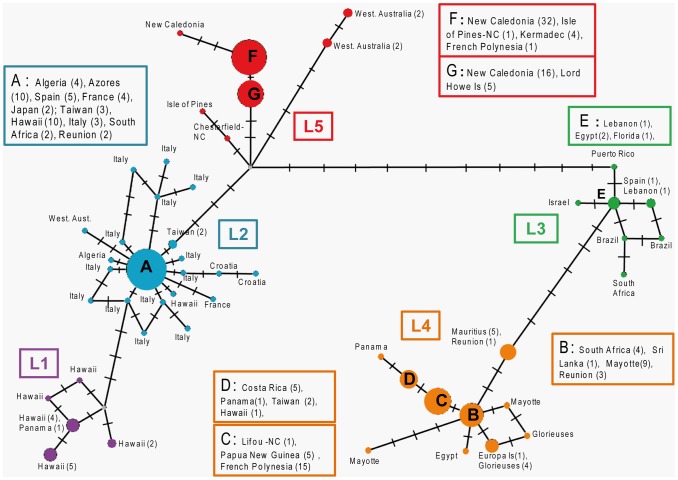
Haplotype network based on the cox2-3 spacer alignment for *A. taxiformis*. L1 is shown in purple, L2 in blue, L3 in green, L4 in yellow and L5 in red.

The network analysis was restricted to *A. taxiformis* due to the large difference in sequences between *A. armata* and *A. taxiformis* that prevents accurate interpretation of the network. In addition, we were mainly interested in examining the spatial distribution of the cryptic taxa revealed in our study with respect to those studied previously. Fifty cox2-3 haplotypes were found within *A. taxiformis*. The haplotypic network illustrates the clustering observed in the phylogenetic trees and the existence of two major groups (L1-L2-L5 and L3-L4) separated by 11 mutation steps (Figure 5).

Lineage 2 was the most diverse and reticulated lineage with 21 haplotypes. Lineages L1 and L3 were the least diverse lineages with 5 and 7 haplotypes respectively, although this low diversity can be attributed to the lower number of specimens analyzed for these two lineages.

Considering the geographical distribution of these lineages, L1 to L4 did not mirror any biogeographical unit or other spatial organization: they were found over a very large spatial scale encompassing both hemispheres and several oceans (Figures 1 and 5). Conversely, results for L5 indicated a much more restricted distribution. L5 is indeed found in Western Australia, Kermadec Island, New Caledonia, Lord Howe Island and Gambier Island (French Polynesia): i.e., in the southern Indo-Pacific.

### Herbarium data

Only nine sequences for the cox2-3 spacer were obtained from the 17 herbarium samples, possibly because of damaged DNA as a result of preservation techniques (air-dried specimens). The Lifou Island sample appeared to belong to L4, while all other samples were assigned to L5. The cox2-3 spacer sequences were available for specimens obtained from a recent sampling expedition and herbarium collection in one location only, Koumac: all the samples belonged to L5 and shared the same cox2-3 haplotype.

## Discussion

### Increasing taxonomic complexity within the genus *Asparagopsis*

*Asparagopsis taxiformis* and *A. armata* were clearly distinguished from each other with the mitochondrial cox2-3 spacer, the nuclear LSU marker and the chloroplastic rbcL spacer. Corroborated by recognized morphological differences [13], [15], [55], this molecular differentiation [at three different markers] highlights the long evolutionary divergence between the two taxa. Our study however brought significant new insights into the cryptic molecular diversity of the genus *Asparagopsis* and documented the existence of two new genetic clusters within each of the two taxonomically accepted species *A. taxiformis* and *A. armata*. This brings the total number of distinct entities to seven across the two taxa.

Ni Chualáin et al. [12] were the first to demonstrate the existence of different genetic clusters within the genus *Asparagopsis* using plastid DNA RFLPs. They identified two clusters within *A. taxiformis*, with disjoint and large distributions: one distributed across the Pacific and Mediterranean Sea and the other spanning the Caribbean and found around the Canary Islands. Using the mitochondrial cox2-3 spacer, Andreakis et al. [3] then separated the two *A. taxiformis* clusters into four lineages with no changes to previous findings regarding *A. armata*. The three markers used in our study showed two well-supported and mutually monophyletic clades within *A. armata*, characterized by a high level of divergence (D = 7.25% for the cox2-3 spacer, 3.23% for LSU and 3.16% for the rbcL spacer). For each marker, data were consistent with divergence levels usually observed among species in Rhodophyta, although divergence rates may vary among species and genera. For example, divergences among species of the Gelidiales are 3.1–11.5% and 0.4–2.2% for the rbcL spacer and LSU, respectively [56], and divergence for the cox2-3 spacer between two species of the genus *Peyssonnelia* is 5.8–6.7% [57]. Given that all three markers showed that there are two separate clades indicates that hybridization has not recently or often occurred between the two clades. We thus suggest that the *A. armata* clades are in fact two cryptic biological species. This hypothesis is reinforced by the fact that the new clade of *A. armata* is restricted to Western Australia, Tasmania and New Zealand based on our sampling. In Australia, both monoecious and dioecious gametophytes have been described [29]. One possibility to be explored is that the two clades may be distinguished by their reproductive features.

Regarding *A. taxiformis*, we observed a new lineage not described in previous studies [3] with specimens collected in the South Pacific (New Caledonia, Lord Howe Island, Kermadec Islands and Gambier Islands in French Polynesia) and Western Australia. Our data could not resolve the taxonomic status of the five lineages within *A. taxiformis*. The molecular differences observed among these lineages at the cox2-3 spacer were however much lower than between *A. taxiformis* and *A. armata*, suggesting recent divergence. In addition, the mitochondrial lineages were often disparate from the nuclear and chloroplastic markers. Such inconsistencies across markers have previously been described for the Gelidiales [58] and the genus *Sargassum* [59] and can be attributed to recombination events between taxa that are not fully reproductively isolated. Using the cox2-3 spacer, the different levels of divergence among lineages within *A. taxiformis* may be indicative of successive separation events with a more ancient divergence between the two clusters [L1-L2-L5] *versus* [L3-L4] (D = 4.88%; around 5.40 Ma; Figure 4) than within the clusters (e.g. between L1 and L2, D = 1.93% with a divergence time around 1.75 Ma). Divergence times were based on the molecular clock obtained by Zuccarello and West [50] for the genus *Bostrychia*, which was estimated using the time of closure of the Isthmus of Panama. As no fossil data are available for *Asparagopsis*, proper calibration of the date for clade separation is not possible and divergence estimates must be considered with caution. Calibration based on the age of paleogeographic events is considered as the most trustworthy; it nonetheless assumes that every lineage evolves at the same substitution rate, which is still open to debate [60].

### Biogeography of *Asparagopsis* in a changing world

Regarding their large spatial distribution, at a first glance, *A. taxiformis* and *A. armata* could be considered as cosmopolitan species. Molecular approaches have often shown that a single species spread across several biogeographic regions hide cryptic species, each of them often located over non-overlapping, discrete geographic ranges [61]. For example, a kelp previously named *Lessonia nigrescens* and found across two biogeographic provinces along the Chilean coasts is in fact composed of two parapatric species, now recognized as *L. berteroana* and *L. spicata* [62], each distributed in a single biogeographic province. Also, the widespread cnidarian *Aurelia aurita* turns out to be a complex of seven sibling species [63]. There are many other examples in the literature for algae [64], polychaetes [65], tunicates [66] and gastrotrichs [67]. *A. armata* and *A. taxiformis* are both composed of several lineages or clades, all defined based on their genetic cohesiveness and characterized by large divergence times (>2 My for the closest ones, L2 and L5). However, only a few lineages were restricted to a single biogeographic region (e.g. L5, see details below). Considering the limited innate dispersal ability of *Asparagopsis* spp., the fact that most of the lineages are distributed across several oceans and/or hemispheres means that their actual distribution may be driven by one (or several) human-mediated introduction process(es).

The different lineages in *A. taxiformis* showed three different distribution patterns. First, L5 perfectly matched a distinctive region in the South Pacific (New Caledonia, Lord Howe Island, Kermadec Islands and Gambier Islands in French Polynesia) and Southwest Australia. In addition, samples from Southwest Australia were genetically more distant from the other samples, closely following the geographical barrier between the Indian and Pacific oceans. This distribution is consistent with previous phycological research that shows biogeographical affinities between marine flora from high latitude areas and zones of the South Pacific [68], [69]. Second, L2 and L3 both showed a large distribution across several biogeographic areas; the observed pattern cannot be maintained by natural dispersal and gene flow at such a broad scale (e.g., some L2 individuals in the Mediterranean Sea were genetically identical to individuals from Hawaii); the low molecular divergence within L2 and within L3 associated with their extensive distribution around the world are typical features of species introduced on a worldwide scale. Their native distribution range is unknown and the two lineages may be cryptogenic lineages (*sensu* Carlton [9]). L2 and L3 fall in the long list of macroalgal species distributed at a global scale due to human-mediated transport (e.g., *Caulerpa taxifolia* and *C. racemosa* from Australia [70]–[74] through the aquarium trade, *Undaria pinnatifida* from Asia via aquaculture and marine traffic [7]). Third, L1 and L4 are intermediate cases. L1 seems to have a natural distribution: it was mostly described in Northeastern Pacific. One record mentions its presence in the South Pacific in French Polynesia, but with high divergence from the Hawaiian and Panamanian samples of the same study [54]. The precise sampling locality is unknown (J. Bolton, pers. com.). We did not recover any L1 during our surveys of the Polynesian islands. Its record in French Polynesia may thus be either a cryptic introduction from the northern hemisphere that failed to establish or a new (sub)lineage in a location that we did not survey. L4 also shows a distribution nearly compatible with a natural distribution: it is found in the Indo-Pacific regions, more specifically in the Coral Triangle (Papua New Guinea) as well as in the Pacific and Indian Oceans and in the Red Sea, which is typical of many species distributed in the Indo-Pacific [75]. It is noteworthy that some haplotypes are restricted to either the Pacific (H7, H16 and H43 in [3]) or the Indian (H5, H10, H11, H20, H21 and H22) Ocean, suggesting a more recent evolutionary divergence on either side of the Coral Triangle, a pattern also described in some mollusks [75]. The presence of L4 in some disconnected and remote locations (e.g. Panama, Costa Rica) most likely results from long-distance dispersal events through human-mediated transport.

The *Asparagopsis* genus clearly illustrates how difficult it is to analyze biogeographic patterns in a changing world, because human-mediated, long-distance dispersal events are disrupting the natural footprints left by historical vicariant events. Reconstructing pathways and vectors of introductions in *Asparagopsis* spp. was not the goal of this study and sampling was not adequate for addressing this issue. However, we could nonetheless differentiate between native and introduced status of L5 in New Caledonia. When we started this study, the worldwide invasive lineage L2 was expected to be present in New Caledonia, where it was reported by Andreakis et al. [3] who sequenced one specimen. The presence of the “invasive” L2 conveniently explained the algal blooms observed in this region. However, in our study, we sequenced the same specimen (voucher IRD no. 10832), which appeared to belong to the new L5 lineage. Sequences obtained from various herbarium specimens collected within the last decade suggested that L5 has remained stable through time, at least in the time frame covering 2001–2013. Based on its well-defined spatial distribution and genetic cohesiveness (i.e., low genetic divergence within L5), L5 is not likely to have been introduced in the South Pacific. The observed blooms are thus more likely due to environmental disturbance than to invasion. In other words, they are more likely to be similar to the green tides of *Ulva* and *Enteromorpha* observed in Europe due to eutrophication [76], [77] or to reef overgrowth by Fucales (*Sargassum* and *Turbinaria* genera) [78], [79]. Another interesting point is that documented cases with intermingled lineages are extremely rare (Table S1). In Hawaii, L2, L1 and L4 co-occur [11], but seem to be distributed in different reef habitats (A. Sherwood, pers. com.; see also Figure 4 in [11]). Either niche partitioning (with local adaptation to micro-habitats) or competitive exclusion between lineages makes syntopy rare. This issue deserves further experimental ecology studies and field surveys, accompanied by accurate lineage identification.

### The critical role of sampling strategy in biogeography, phylogenetic and invasion studies

Although previous studies provided results on widely distributed samples [3], [10], [11], [24], [54], our study showed additional lineages and even probable cryptic species, in *A. armata* in particular, by expanding sampling range and size. For *A. armata*, the material considered by Andreakis et al. [3] came from European localities only. This may explain the apparent molecular uniformity and the identification of only one clade in their study, contrasting with our study in which samples were collected in several new regions. Our results demonstrated the importance of targeting surveys in areas where the species may be distributed, including some remote and overlooked locations, to identify new operational evolutionary units and avoid bias in the estimation of haplotypic richness. Broadly speaking, the estimation of the magnitude of marine species richness depends greatly on sampling effort [80]. For example, both the haplotype and species richness of rotifers in Polish lakes are positively correlated with the number of samples and the number of localities sampled [81]. Sampling effort has also long been recognized as important in the study of invaders [82], [83]. There are three categories of errors that can underestimate the number of invaders: 1) systematics, 2) biogeography, and 3) sampling [5]. The case of *Asparagopsis* matches that typology. First, with regard to systematics, the genus *Asparagopsis* is composed of complexes of cryptic species, and new introduced taxa can easily be missed. Secondly, *Asparagopsis* spp. biogeography is difficult: *A. taxiformis* was first described in Alexandria in the Mediterranean Sea. This type locality was thus falsely reported as being in the native range. Likewise, *Neosiphonia harveyi* ( = *Polysiphonia harveyi*) was first described in 1848 using North American samples (Connecticut) and shown to be native to Asia 140 years later [84]. Finally, by significantly expanding sampling coverage compared to previous studies, we discovered the presence of lineages that may have been introduced (e.g., L2 in Reunion Island).

**Figure 6:**
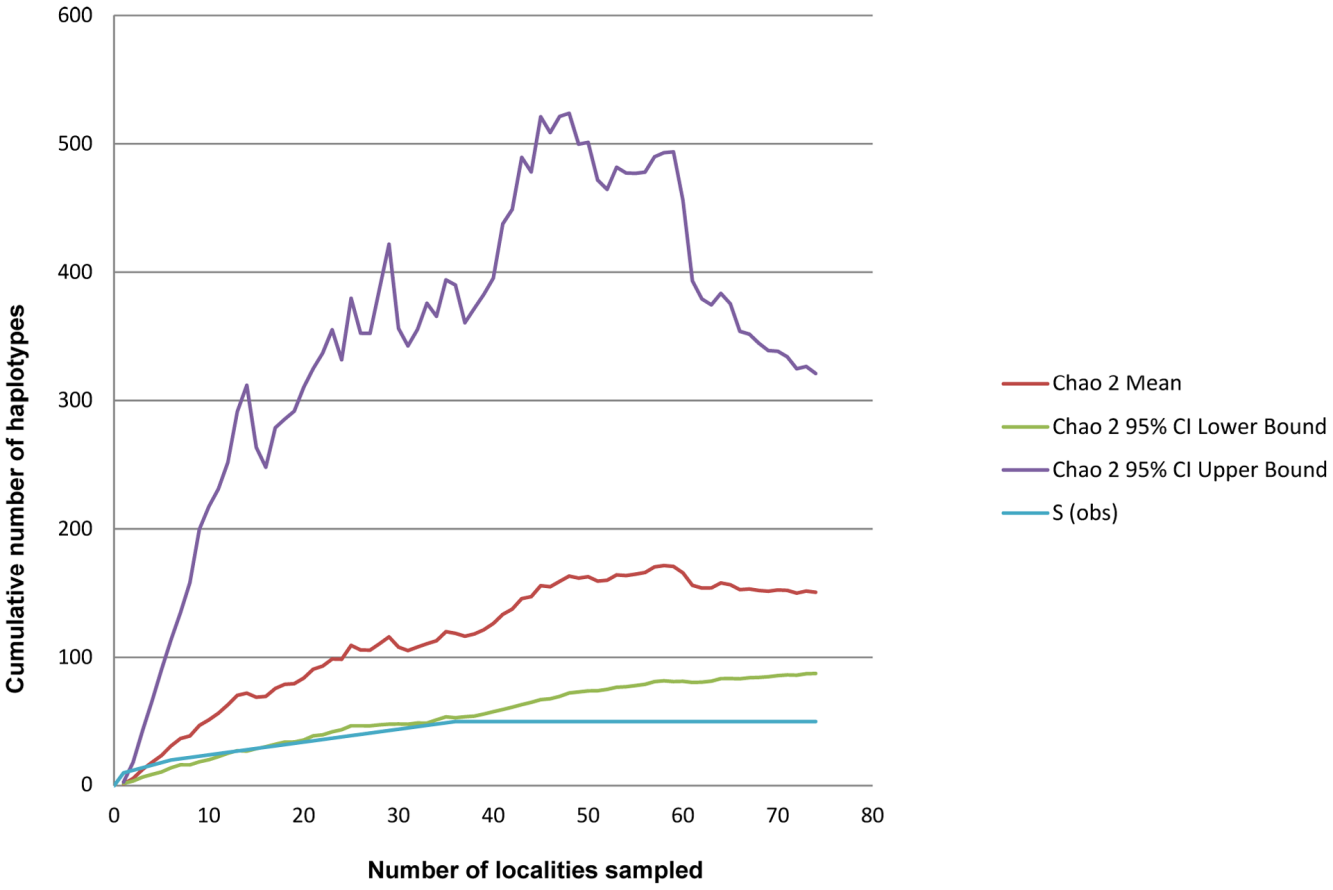
Estimate of haplotype richness (Chao2) for *Asparagopsis taxiformis* against the number of localities sampled.

Nevertheless, *Asparagopsis* sampling effort may still not be sufficient. Our findings were based on specimens from a few relatively comprehensively sampled areas (e.g., Mediterranean Sea [10], [12], [24], Hawaii [11], New Caledonia, this study) and a few samples from other areas (e.g., four samples from South Africa [54], one sample from New Zealand, this study). Given the invasive nature of *Asparagopsis* and its cryptic diversity, an optimal sampling strategy should include a two-pronged approach: large geographical coverage with a substantial number of specimens from each site. To test the accuracy of our sampling, we used ESTIMATES software [85] to compute the expected haplotype number using the non-parametric estimator Chao 2. This estimator draws its inferences on singletons and private haplotypes. Plotting this estimator and the observed number of haplotypes with respect to the number of localities (Figure 6), we observed a number of haplotypes well below the lowest estimate of the expected number of haplotypes (for 74 localities, only 50 haplotypes were observed compared to the expected number of between 87 and 321 haplotypes). Thus, despite an ample sampling effort, we likely underestimated the true haplotypic richness of *A. taxiformis*. Thus, further sampling of Central and South Pacific is necessary to establish a more accurate distribution of L5 and to collect specimens in the Coral Triangle, poorly investigated so far, although it is a center of diversity and diversification [86], [87] in the Indo-Pacific region.

In conclusion, our findings illustrate the importance of comprehensive sampling including in overlooked regions, when examining diversity at the inter- and intra-taxon level. The delineation of operational evolutionary entities (genetically cohesive lineages) and mapping their distribution can help distinguish between native and introduced lineages. In introduced lineages, the biogeographic identity of the lineage has been disrupted (e.g. L2) by human-mediated transport, whereas it has been maintained in native lineages (e.g., L5). This pattern of introduced vs. native lineages is discernable if gene flow between the two ranges has not been interrupted or if the time elapsed since the introduction is not too long. Accordingly, we clearly demonstrated that several cryptic taxa, some being introduced, explain the apparent cosmopolitanism of the *Asparagopsis* genus. A meticulous molecular examination of herbarium specimens can help assess the consistency of taxa through times. Cryptic diversity was observed at various taxonomical levels, as illustrated by two highly divergent clades within *A. armata*, and five more or less divergent lineages within *A. taxiformis*. Integrative taxonomy studies [88]–[91] with further phylogenetic analyses, biogeography studies and morphological taxonomy work as well as cross-fertilization experiments are all good means to test for contemporary reproductive isolation among lineages, and investigate their status as separate biological species. This need of taxonomic revision is also supported by recent evidences of morphological variability among lineages [92].

## Supporting Information

Figure S1 **Detailed neighbor-joining tree and Bayesian analysis for the cox2-3 spacer.** Posterior value from Bayesian analysis are given. Specimen's name is explained in Table S1.
(TIF)

Figure S2 **Detailed neighbor-joining tree for the rbcL spacer.** Bootstrap values are given. Specimen's name is explained in Table S1.
(TIF)

Figure S3 **Detailed neighbor-joining (NJ) tree for LSU.** Bootstrap values are given. Specimen's name is explained in Table S1.
(TIF)

Table S1 **List of the specimens analysed.** Details are provided concerning their origin, species, cox2-3 lineage identified and their GenBank accession numbers for each of the marker sequenced.
(XLS)
