# Supplementary figures and images for: The More We Search, the More We Find: Discovery of a New Lineage and a New Species Complex in the Genus Asparagopsis
Source: PLoS One. 2014 Jul 30;9(7):e103826. doi: 10.1371/journal.pone.0103826 (PMC4116237; doi:10.1371/journal.pone.0103826)

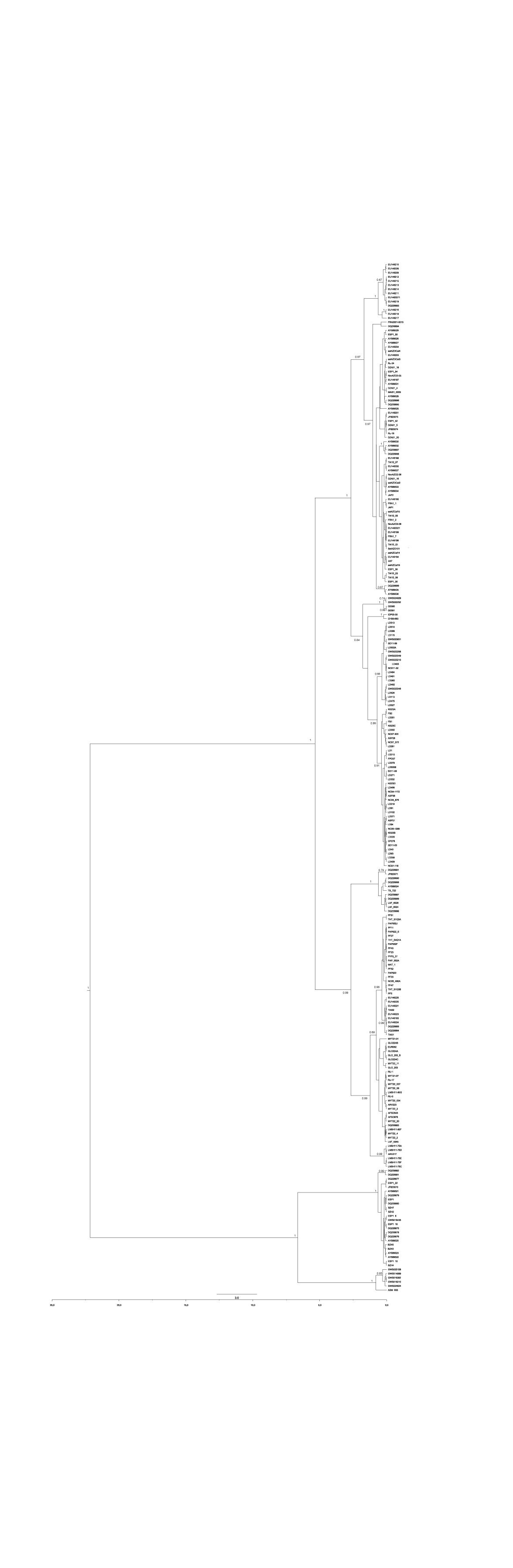

Supplement: Figure S1 — Detailed neighbor-joining tree and Bayesian analysis for the cox2-3 spacer. Posterior value from Bayesian analysis are given. Specimen's name is explained in Table S1. (TIF) [file pone.0103826.s001.tif]

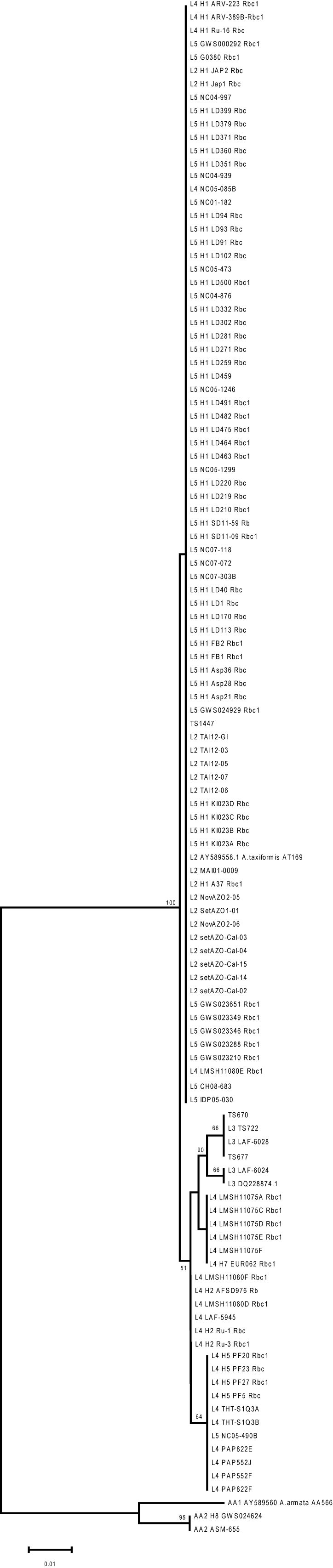

Supplement: Figure S2 — Detailed neighbor-joining tree for the rbcL spacer. Bootstrap values are given. Specimen's name is explained in Table S1. (TIF) [file pone.0103826.s002.tif]

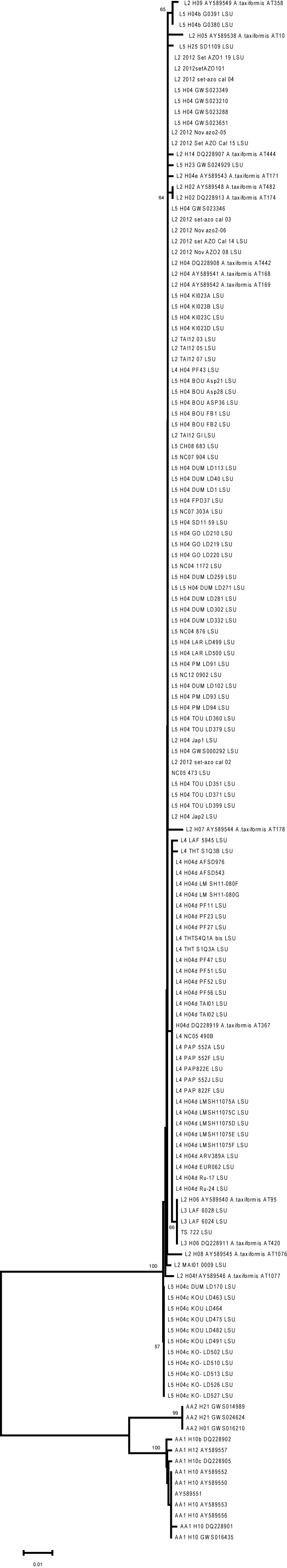

Supplement: Figure S3 — Detailed neighbor-joining (NJ) tree for LSU. Bootstrap values are given. Specimen's name is explained in Table S1. (TIF) [file pone.0103826.s003.tif]
